# Characteristics of Acupuncture Treatment Associated with Outcome: An Individual Patient Meta-Analysis of 17,922 Patients with Chronic Pain in Randomised Controlled Trials 

**DOI:** 10.1371/journal.pone.0077438

**Published:** 2013-10-11

**Authors:** Hugh MacPherson, Alexandra C. Maschino, George Lewith, Nadine E. Foster, Claudia Witt, Andrew J. Vickers, on behalf of the Acupuncture Trialists' Collaboration

**Affiliations:** 1 Department of Health Sciences, University of York, York, United Kingdom; 2 Memorial Sloan-Kettering Cancer Center, New York, New York, United States of America; 3 University of Southampton, Southampton, United Kingdom; 4 Arthritis Research UK Primary Care Centre, Institute of Primary Care and Health Sciences, Keele University, Keele, United Kingdom; 5 Institute for Social Medicine, Epidemiology and Health Economics,Charité Universitätsmedizin, Berlin, Germany; The James Cook University Hospital, United Kingdom

## Abstract

**Background:**

Recent evidence shows that acupuncture is effective for chronic pain. However we do not know whether there are characteristics of acupuncture or acupuncturists that are associated with better or worse outcomes.

**Methods:**

An existing dataset, developed by the Acupuncture Trialists’ Collaboration, included 29 trials of acupuncture for chronic pain with individual data involving 17,922 patients. The available data on characteristics of acupuncture included style of acupuncture, point prescription, location of needles, use of electrical stimulation and moxibustion, number, frequency and duration of sessions, number of needles used and acupuncturist experience. We used random-effects meta-regression to test the effect of each characteristic on the main effect estimate of pain. Where sufficient patient-level data were available, we conducted patient-level analyses.

**Results:**

When comparing acupuncture to sham controls, there was little evidence that the effects of acupuncture on pain were modified by any of the acupuncture characteristics evaluated, including style of acupuncture, the number or placement of needles, the number, frequency or duration of sessions, patient-practitioner interactions and the experience of the acupuncturist. When comparing acupuncture to non-acupuncture controls, there was little evidence that these characteristics modified the effect of acupuncture, except better pain outcomes were observed when more needles were used (p=0.010) and, from patient level analysis involving a sub-set of five trials, when a higher number of acupuncture treatment sessions were provided (p<0.001).

**Conclusion:**

There was little evidence that different characteristics of acupuncture or acupuncturists modified the effect of treatment on pain outcomes. Increased number of needles and more sessions appear to be associated with better outcomes when comparing acupuncture to non-acupuncture controls, suggesting that dose is important. Potential confounders include differences in control group and sample size between trials. Trials to evaluate potentially small differences in outcome associated with different acupuncture characteristics are likely to require large sample sizes.

## Introduction

A recent individual patient data meta-analysis conducted by the Acupuncture Trialists' Collaboration provided clear evidence that acupuncture is of benefit for chronic pain[1]. The study included close to 18,000 patients with one of four chronic pain conditions: osteoarthritis, headache, back and neck pain, shoulder pain. Comparisons were made between acupuncture and sham acupuncture, as well as between acupuncture and a range of non-acupuncture controls, such as wait list and usual care. Benefits in terms of effect sizes (standardised mean differences) were in the order of 0.15 to 0.23 for the comparison between acupuncture and sham and 0.5 for the comparison with non-acupuncture controls[1]. 

Acupuncture is not a single standardized intervention. In routine clinical practice, the same patient may receive acupuncture with different characteristics from different practitioners. These differences include specific characteristics of treatment - such as the number and frequency of sessions or the additional use of electrical stimulation - as well as the overall “style” of acupuncture. A distinction is often made between traditional Chinese acupuncture, in which diagnosis and treatment are based on a theoretical framework involving patterns of symptoms and concepts such as yin, yang and the strength of qi, and Westernized approaches, involving a neuro-anatomical basis for diagnosis and treatment[2]. The estimates of effect sizes such as those reported by the Acupuncture Trialists' Collaboration are averages across different acupuncturists and styles of acupuncture[1]. It is natural to ask, therefore, whether there are characteristics of acupuncture style or acupuncturists that are associated with patient outcome, that is, whether there are modifiers of acupuncture effect.

Assessing effect modification in clinical trials is associated with considerable power concerns [3,4]. Trials are usually designed with only sufficient power to test the primary hypothesis in which case they will be inherently underpowered to determine whether individual characteristics of treatment might be associated with affect outcome. The sample size needed to detect an interaction has to be many times greater than that needed to detect a primary effect[5]. Combining summary data from a number of trials in a meta-analysis can overcome the problem of limited power to some extent. An individual patient data meta-analysis is more powerful as it allows patient-level analysis[6]. 

The large individual patient dataset developed by the Acupuncture Trialists Collaboration[1] incorporates raw data from high quality randomised controlled trials of acupuncture for chronic pain. In this study, we analyse this dataset to determine whether there are characteristics of acupuncture or acupuncturists that act as effect modifiers for acupuncture treatment outcome.

## Methods

### Included Trials

Trials included in these analyses were identified through a systematic literature review that has been previously described[1]. The search included trials of acupuncture for chronic pain published prior to November 2008 and included only trials where allocation concealment was determined unambiguously to be adequate. Eligible pain types were non-specific back or neck pain, shoulder pain, chronic headache or osteoarthritis - with the additional criterion that the current episode of pain must be of at least four weeks duration. This search resulted in the identification of 31 trials. 

### Data Acquisition

Individual patient data were obtained from 29 trials. Data on the trial-level characteristics of the acupuncture intervention were obtained directly from responding trialists by use of a questionnaire and are presented in Appendix S1.

### Outcome

The primary outcome used for this analysis was pain as defined as primary outcome by the responding author of each study. Where multiple criteria were considered in the primary outcome (e.g. a response defined as either a 33% reduction in pain or a 50% reduction in pain medication) or if the primary outcome was inherently categorical, we used a continuous measure of pain measured at the same time point as the original primary outcome. To make the various outcome measurements comparable between different trials, the primary endpoint of each was standardized by dividing by pooled standard deviation. 

### Acupuncture Characteristics

The characteristics of acupuncture we investigated included the style of acupuncture, the point prescription, the location of needles, the use of electrical stimulation and moxibustion, whether acupuncture-specific patient practitioner interactions were allowed, acupuncturist experience, the number, frequency and duration of sessions, and the number of needles used. Some trials recorded patient-level data on certain acupuncture characteristics, such as the number of sessions, the duration of sessions and the number of needles used. In these cases a summary statistic was calculated and entered into analysis. If no such data were available, the values reported by trialists on the data abstraction sheets were used. 

#### Style of acupuncture

Trialists defined the style of acupuncture as based on traditional Chinese theory, or contemporary Western acupuncture or a mixture of both approaches. For our main analysis, we compared trials that used at least some traditional approaches to those where the acupuncture was purely Western; as a sensitivity analysis, we compared trials using purely a Chinese traditional approach to those that used at least some Western techniques. 

#### Point Prescription

The point prescription was defined as fixed, flexible or individualized. Responding authors of the trials were asked to select from these three options. Trials were categorized as flexible needle formula if trialists indicated that acupuncture was semi-standardized, flexible with fixed points, or both fixed and flexible formulas. The analyses compare flexible and individualized formula to the reference group, fixed needle formula. 

#### Stimulation

Trialists were asked to report if their trial allowed electrical stimulation to be added to the needles during acupuncture sessions.

#### Moxibustion

Trialists were asked to report if acupuncturists were allowed to prescribe moxibustion.

#### De Qi

Trial level information on de qi needle sensation was collected. If acupuncturists attempted to elicit de qi, whether felt by acupuncturist or patient, then the trial was considered to have attempted de qi. 

#### Acupuncture-specific patient practitioner interactions

Acupuncture-specific interactions between the patient and the acupuncturist can occur through explanations of treatment, advice, support, and suggestions about helpful lifestyle changes, which are driven by acupuncture theory and intended to influence outcome. These non-needling aspects of treatment can be considered integral to and characteristic of acupuncture. Trialists were asked if their trial allowed or encouraged these interactions and associated patient self-help actions. If the trialist replied that these kinds of interactions were not prohibited at the trial level it was assumed that they might have taken place since they are often part of standard practice. 

#### Minimum years of practice required

Trialists were asked to report the minimum number of years of practice as an acupuncturist required to participate in their trial. If trialists did not indicate a requirement, the trial was included in the analysis as having zero years required, even if trialists noted that all acupuncturists were experienced. Otherwise, the minimum number of years required was entered into the analyses as a continuous variable. 

#### Maximum Number of sessions allowed

The maximum number of acupuncture treatment sessions allowed during the trial period was reported by each trialist. If participants were allowed extra sessions pending partially successful treatment, these were not counted towards a maximum since it was frequently the case that extra sessions were only offered to partial responders. If patient-level data were available an average was taken. Data were analyzed per 5 session increments. 

#### Frequency of sessions

The frequency of sessions was recorded and analyzed continuously, as a weekly average (ie. typical number of sessions per week). If a range was given, or if the frequency varied over the study period, the mid-point was taken. 

#### Duration of sessions

The duration of sessions was reported as the average length of a session in minutes among the patients receiving acupuncture. The mid-point was used if a range was given. We used average needle retention time for two trials that reported this characteristic but not overall treatment session time. Patient-level data were used where available by taking the mean duration of patients’ sessions. Trials were not included in this analysis if treatment was individualized and no individual level data were available. Duration of sessions was included as a continuous variable in the analyses and results reported per 5 minute increments.

#### Average Number of needles used

Trialists were asked to report the average number of needles used per treatment session. If a range was given, the mid-point was taken. For one trial, the author specified that 9 needles were used in each affected leg. The paper published from this trial reported that 25% of patients in the acupuncture group had both knees affected so therefore this trial was included in the analysis has having an average of 11 needles. Trials were excluded from this analysis if the number of needles was unknown. If patient-level data were available an average was included. The average number of needles used was analyzed as a continuous variable, with the coefficient reported in 5 needle increments. 

#### Location of Needles

The placement of acupuncture needles was categorized as local (at or near the location of pain) or distal to the location of pain), or both. 

### Statistical Methods

Data on effect modifiers were either at the individual or trial level. Using number of treatment sessions as an example, this would constitute individual level data if the number of acupuncture sessions received by each patient had been recorded on the study database, but constitute trial level data if either all patients received the same number of sessions or data were not recorded at the patient level and an estimate of the average number of sessions was provided by the trialists. 

For each trial, we identified the primary outcome defined by the study authors in terms of both the scale and time point. We kept endpoints on the continuous scale. If multiple criteria are considered in the primary outcome, or if the primary outcome is inherently categorical, we will use a continuous measure of pain measured at the same time point as the original primary endpoint. For analyses that include trials with different primary endpoints, we will create a standardized primary endpoint by dividing by standard deviation. 

For trial-level analyses, we used random-effects meta-regression to test the effect of each characteristic on the main effect estimate using the Stata command *metareg*. We first calculated the effect size and standard error for each trial as described in our main paper[1]. For each treatment characteristic under study, we then entered the effect size and standard error for each trial into a meta-regression along with the trial level average for that characteristic. The coefficients obtained from these analyses are estimates, in standard deviations, of the effect of each acupuncture characteristic on the main treatment effect. Using session time to illustrate, this analysis addresses questions about effect modification in the form of: “Do trials where the practitioners provided patients with longer session times on average show larger or smaller effects of acupuncture than trials where the average session time was shorter?”

Patient-level analyses included the number of sessions, the number of needles, and the age and gender of the acupuncturist. For each trial, we created a linear regression as for the main analysis of effect size, but included the characteristic and an interaction term between the characteristic and treatment allocation. The coefficient and standard error for the interaction term represents the change in the outcome score in standard deviations associated with the acupuncture characteristic in the acupuncture treatment group. This was then entered into a meta-analysis, using the Stata command *metan*. The interpretation of the results is similar as to the results from the trial level analysis. In both analyses, random effects estimates were used. 

A pre-planned sensitivity analysis excluded a set of outlying trials, all by the same team, which had very much larger effect sizes than other trials. This sensitivity analysis has previously been conducted in the primary analysis of effect size[1]. 

Analyses were conducted separately for sham and non-acupuncture controls. All analyses were conducted using Stata 12.0 (Stata Corp., College Station, TX). 

## Results

Characteristics of each individual trial are presented in Appendix S1. Table 1 provides a summary of the trial-level characteristics, while Table 2 provides a summary of patient-level characteristics. In a majority of trials the acupuncture was based on traditional Chinese acupuncture (59%) and had a flexible point prescription (55%). In all 29 trials manual needle stimulation was used in the acupuncture group, while only about quarter of trials allowed the addition of electrical stimulation (n=7) and 14% allowed moxibustion (n=4). Attempts to elicit de qi in the acupuncture group were made in all 25 trials that provided this information. The maximum number of sessions varied widely, from 3 to 30, as did the mean number of needles used (range 1-18) and the mean session duration (range 15 to 32 minutes). The mean session frequency ranged from one session every eight days to two sessions a week. 

**Table 1:** Trial-level acupuncture characteristics. Frequency (%). N=29.

**Style of acupuncture**	
Traditional Chinese techniques	17 (59%)
’Western’	4 (14%)
Combination of traditional Chinese and Western	8 (28%)
**Point Prescription**	
Fixed needle formula	4 (14%)
Flexible formula	16 (55%)
Individualized	9 (31%)
**Location of needles**	
Local Points Only	0 (0%)
Distal Points Only	1 (3%)
Both Local and Distal Points	28 (97%)
**Electrical stimulation allowed**	7 (24%)
**Moxibustion allowed**	4 (14%)
**De Qi attempted (n=25)**	25 (100%)
**Acupuncture-specific patient practitioner interactions**	12 (41%)
**Minimum years of experience required**	
No requirement specified (0 years)	12 (41%)
6 months to 2 years	5 (17%)
3 years	9 (31%)
5 years	2 (7%)
10 years	1 (3%)
**Maximum number of sessions**	
3-5	3 (10%)
6-10	14 (48%)
11-15	7 (24%)
16-20	3 (10%)
21-30	2 (7%)
**Frequency of sessions (mean number of sessions per week)**	
0.88	1 (3%)
1	14 (48%)
1.5	7 (24%)
1.67	1 (3%)
2	6 (21%)
**Mean duration of sessions, rounded to whole numbers (n=26)**	
15-19 minutes	1 (4%)
20-24 minutes	4 (16%)
25-29 minutes	6 (24%)
30+ minutes	14 (56%)
**Mean number of needles used, rounded to whole numbers (n=25)**	
1-4	1 (4%)
5-9	6 (25%)
10-14	9 (38%)
15-20	8 (33%)

**Table 2:** Patient-level acupuncture characteristics. Frequency (%). N=18,434.

**Number of Sessions**	
0	383 (2%)
1-5	402 (2%)
6-10	7161 (39%)
11-15	1998 (11%)
16-20	45 (<1%)
21-30	16 (<1%)
Missing	1806 (10%)
Not reported	6623 (36%)
**Average Session Duration**	
2-15	166 (1%)
15-30	2552 (14%)
31-45	406 (2%)
46-60	60 (<1%)
60+	3 (<1%)
Missing	1257 (7%)
Not reported	13990 (76%)
**Average Number of Needles**	
2-5	20 (<1%)
6-10	610 (3%)
11-15	717 (4%)
16-20	627 (3%)
21-25	177 (1%)
26+	27 (<1%)
Missing	2529 (14%)
Not reported	13727 (74%)
**Age of Physician/Acupuncturist**	
30-35	298 (2%)
36-40	2119 (11%)
41-45	2630 (14%)
46-50	2407 (13%)
51-55	1701 (9%)
56-60	872 (5%)
60+	303 (2%)
Missing	368 (2%)
Not reported	7736 (42%)
**Male Physician/Acupuncturist**	
Male	7002 (66%)
Female	3626 (20%)
Missing	0 (0%)
Not reported	7806 (42%)

In the trial-level data we did not find evidence that any of the acupuncture characteristics evaluated, including style of acupuncture, the number or placement of needles, the number, frequency or duration of sessions, patient-practitioner interactions or the experience of the acupuncturist, significantly changed the effect of acupuncture on pain (all p>0.05) in sham controlled trials (Table 3). We also found little evidence that these characteristics modify the effect of acupuncture when acupuncture was compared to non-acupuncture controls. The exception was that acupuncture effects increased in comparison to non-acupuncture controls when more needles were used (increase in effect size per 5 needles of 0.33; 95% CI 0.08, 0.58; p=0.010).

**Table 3:** Results of univariate meta-regression analyses.

	***ACUPUNCTURE VS. SHAM n=20***			***ACUPUNCTURE VS. NON- ACUPUNCTURE n=18***		
	β*	95% CI	P value	β*	95% CI	P value
**Style of acupuncture**						
Some TCM v Western only	0.05	-0.52, 0.63	0.9	0.13	-0.51, 0.77	0.7
TCM only v Some Western	0.20	-0.20, 0.61	0.3	-0.10	-0.38, 0.19	0.5
**Point Prescription**						
Fixed needle formula		*ref*			*ref*	
Flexible formula	-0.08	-0.58, 0.43	0.8	0.02	-0.64, 0.68	>0.9
Individualized	-0.15	-1.16, 0.86	0.8	-0.08	-0.74, 0.59	0.8
**Electrical stimulation allowed**	0.34	-0.13, 0.80	0.15	-0.19	-0.56, 0.17	0.3
**Manual stimulation allowed**	*all allowed*			*all allowed*		
**Moxibustion allowed**	*all did not allow*			-0.28	-0.63, 0.06	0.11
**De Qi attempted**	*all allowed*			*all allowed*		
**Acupuncture-specific patient practitioner interactions allowed**	-0.22	-0.70, 0.26	0.4	0.06	-0.23, 0.35	0.7
**Minimum experience required (years)**	0.01	-0.08, 0.10	0.8	0.05	-0.05, 0.16	0.3
**Maximum number of sessions (per 5 sessions)**	-0.14	-0.37, 0.08	0.2	0.02	-0.07, 0.12	0.6
**Frequency of sessions (per week)**	-0.19	-0.66, 0.27	0.4	0.09	-0.31, 0.49	0.7
**Duration of sessions** (**per 5 minutes**)^a^	-0.10	-0.30, 0.11	0.4	-0.01	-0.26, 0.24	0.9
**Number of needles used** (**per 5 needles**)^b^	-0.17	-0.37, 0.03	0.095	0.33	0.08, 0.58	0.01

* β is an estimate of the change in the effect of acupuncture in terms of standardized difference compared to controls for each characteristic; a positive β indicates a larger effect of acupuncture compared to controls for trials with the given the characteristic versus the referent level of the characteristic.

a 4 trials excluded missing data

b 5 trials excluded missing data

The results of the sensitivity analysis excluding three outlying trials, which were all sham controlled, all by the same team, and with very much larger effect sizes than other trials, are presented in Table 4. This analysis showed that trials allowing electrical stimulation had a significantly stronger effect of acupuncture compared to sham controls (β= 0.27; 95% CI 0.03, 0.51; p=0.027) and those with a longer average treatment session duration had a smaller effect compared to sham controls (β=-0.14 per 5 minutes; 95% CI -0.22, -0.06; p=0.001).

**Table 4:** Results of meta-regression for acupuncture trials with sham controls, excluding outlying trials.

	***ACUPUNCTURE vs. SHAM n=17***			
	N	Β*	95% CI	P value
**Style of acupuncture**	17			
’Western’ only			*ref*	
Traditional Chinese		-0.14	-0.46, 0.17	0.4
**Point Prescription**	17			
Fixed needle formula			*ref*	
Flexible formula		-0.25	-0.50, 0.00	0.054
Individualized		-0.11	-0.58, 0.36	0.6
**Electrical stimulation allowed**	17	0.27	0.03, 0.51	0.027
**Manual stimulation allowed**	17	*all allowed*		
**Moxibustion allowed**	17	*all did not allow*		
**De Qi elicited (n=26)**	17	*all allowed*		
**Acupuncture-specific patient practitioner interactions allowed**	17	-0.04	-0.28, 0.20	0.8
**Minimum experience required (years)**	17	0.00	-0.05, 0.05	>0.9
**Maximum number of sessions (per 5 sessions)**	17	-0.05	-0.18, 0.08	0.4
**Frequency of sessions (per week)**	17	-0.04	-0.29, 0.21	0.8
**Duration of sessions (per 5 minutes)**	17	-0.14	-0.22, -0.06	0.001
**Number of needles used (per 5 needles)**	17	-0.08	-0.22, 0.05	0.2

* β is an estimate of the change in the effect of acupuncture compared to controls for each characteristic; a positive β indicates a larger effect of acupuncture compared to controls for trials with the given the characteristic versus the referent level of the characteristic.

Patient-level analyses mirrored trial level analyses; the direction of the effect of the acupuncture characteristics was unchanged (Table 5). Importantly, the confidence intervals were much tighter around the patient-level estimates despite the fact that fewer trials could be included in each analysis. For each analysis, there were no more than five trials with patient-level data available for the specific predictor being analyzed. The patient-level analysis for number of sessions in non-acupuncture controlled trials included only 5 trials and 8,292 patients compared to trial-level analysis which included 18 trials with a total of 14,597 patients. The patient-level analysis suggested that a higher number of acupuncture treatment sessions improves the effect of acupuncture (β=0.11; 95% CI 0.01, 0.21; p=0.0007). We considered the possibility of interactions either between for number of sessions and duration of sessions or between the duration of sessions and number of needles, and we found that neither one of these interactions was significantly associated with the effect of acupuncture.

**Table 5:** Results of patient-level analysis of acupuncture characteristics.

	***SHAM***				***NON-ACUPUNCTURE***			
	N	β	95% CI	P value	N	β	95% CI	P value
**Number of sessions (per 5 sessions)**	3 (646/648)	-0.76	-1.75, 0.22	0.13	5 (8292/9321)	0.11	0.01, 0.21	0.0007
**Duration of sessions (per 5 minutes)**	5 (2444/2482)	-0.03	-0.08, 0.03	0.3	less than 3 trials			
**Number of needles used (per 5 needles)**	5 (1769/2484)	-0.11	-0.35, 0.14	0.4	less than 3 trials			
**Age of acupuncturist (per 5 years)**	less than 3 trials				6 (9127/9446)	-0.01	-0.04, 0.02	0.5
**Male acupuncturist**	less than 3 trials				6 (9384/9446)	-0.07	-0.16, 0.02	0.084

N = Number of trials included in analysis (number of patients included in analysis /total number of patients in included trials)

## Discussion

### Principal findings

Our results provide the best information available on treatment effect modifiers in acupuncture trials for chronic pain. For most treatment-related characteristics, we found no evidence of a modifying effect on pain outcomes, including many characteristics that might have been commonly expected to modify outcomes, such as style of acupuncture, use of electrical stimulation, addition of moxibustion, experience of the acupuncturist, and the frequency and duration of sessions. We found that the effect of acupuncture increased in comparison to non-acupuncture controls when more needles were used. This association should be considered in the context of the large number of our hypotheses tested and the rather implausible estimate of an increase in effect size per 5 needles of 0.33, larger than the mean difference between true and sham acupuncture [1]. Additionally, this result is driven by the two trials with the largest positive effect size (Brinkhaus et. al for LBP [7] and Witt et. al 2005 [8] for OA) also used the most needles. These two trials have true waiting list controls while the trial by Foster et al [9], which had the lowest effect size estimate and the lowest number of needles, provided a physiotherapy-led course of advice and supervised exercise to all participants. This evidence-based package of care[10] provided to all trial participants may have led to a ceiling effect making it difficult to identify any additional effect of acupuncture. We also found in a patient level analysis involving a limited dataset from a sub-set of five trials that more treatment sessions was associated with better pain outcomes in acupuncture treatment groups compared to non-acupuncture controls (p<0.001). This suggests that dose of acupuncture could have an impact on treatment outcomes. 

When interpreting these analyses, we carefully considered the results in the context of testing multiple hypotheses. Testing a large number of hypotheses increases the risk of falsely rejecting at least one null hypothesis. Given the largely null results, we did not feel that formal statistical correction to account for multiple testing was justified.

### Comparison with other studies

We did not observe large variations in outcome associated with the style of acupuncture practice. This contrasts with findings from qualitative studies that purported to identify the importance of the theoretical affiliation and institutional context[11,12]. Hughes et al describe how theoretical affiliation can impact on “almost all aspects of treatment”, with “demonstrable implications for the practice and research of acupuncture”.[11] For example, the authors argued that the outcomes from trials of acupuncture “fail to reflect [acupuncture’s] effectiveness in clinical practice”, and hypothesize that therapeutic benefits are likely to be reduced due to an absence of a traditional acupuncture approach that incorporates the notion of “energy work”.[11] Similarly, Paterson and Britten argue that without a theoretical approach to acupuncture that is “holistic”, with an emphasis that includes the process of care, there is likely to be “reduced or absent treatment effects”.[12] These theoretical positions are not supported by our empirical data in patients with chronic pain, although we accept that it is theoretically possible that effect modifiers of acupuncture may be condition specific. 

Our results are in concordance with a pooled analysis of four German-based trials of acupuncture for chronic pain [13], all of which are included in our study. We also found that neither the duration of training or experience as an acupuncturist are associated with an effect of acupuncture. In that previous study, the only physician characteristic that had a significant influence on outcome in the German-based trials was that internists performed slightly better than an average physician and orthopedists slightly worse. As physician specialty was only recorded in these German studies, this analysis was not repeated here. 

Given the results of the primary research [1] showing small differences between real and sham acupuncture, it is not surprising that the current analysis showed little evidence of substantial differences between alternative approaches to acupuncture. Each individual component of acupuncture cannot be expected to make more than a small contribution to any overall effect. 

### Strengths and Limitations

The benefits of an individual patient data meta-analysis as opposed to a meta-analysis of summary data include increased power and the facility to conduct further types of analyses, including the patient level analyses of treatment effect modifiers that we report here. Indeed, it is highly doubtful that questions about effect modification could be addressed outside the context of an meta-analysis. The main limitation, as for any meta-analysis, was data availability. The total number of trials was relatively modest, and analyses with individual patient data included no more than five trials. As a result, many of our analyses had relatively low power, with wide confidence intervals around central estimates. Furthermore, heterogeneity of treatment characteristics was relatively limited. For example, nearly 75% of trials involved between 6 and 15 treatments, and in no trial was acupuncture administered more than twice a week. It is not unusual in China for acupuncture to be given four or five times a week. We were unable to test whether such a level of acupuncture frequency has additional benefit as none of the included primary trials delivered acupuncture this frequently. We also did not have data on syndrome differentiation, a feature that characterizes acupuncture when practiced according to the principles of traditional Chinese medicine, and could therefore draw no conclusions regarding their impact on outcome. And finally, we conducted a large number of tests, and our results must be considered in this context.

### Implications for research

Compared to meta-regression of trial level data, we have shown how individual patient data meta-analysis can dramatically increase the power to explore treatment effect modifiers in patients with chronic pain. We recommend that future trials should record patient level data and make these data available to other researchers. In this meta-analysis, we only included studies published prior to 2008, however the Acupuncture Trialists Collaboration is to incorporate subsequent trials in the individual patient database which will lead to an update of the results. 

There is no reason to believe that contemporary acupuncture trials systematically underestimate treatment effects. Though acupuncturists have long been concerned about what constitutes “correct” practice [14,15], it can be argued that the consensus methods that are often used to determine acupuncture characteristics - number of treatment sessions, duration of sessions, needle prescriptions, and training and experience of acupuncturists - are appropriate. This is because the variations in outcome associated with these factors are likely to be small. We also know that in order to design a trial with sufficient power to detect the small differences in outcome that might be associated with different acupuncture characteristics, very large sample sizes will be needed. This study would suggest that the most useful characteristics to test out would be the number of needles and number of treatment sessions. By way of an example, if we hypothesize that the difference in outcome between two individual acupuncture characteristics of treatment is associated with an effect size of 0.05, then a trial comparing these would require approximately 12,500 patients. 

When exploring practitioner-related effects on outcome, the few characteristics of acupuncturists that were reported sufficiently consistently by trialists, namely age, gender and minimum experience as an acupuncture practitioner, are likely to be too simple. It is possible that there is variation associated with individual practitioners, that some have better results than others. A similar situation has arisen for other therapist-led interventions[16,17]. To better understand the reasons for any heterogeneity between acupuncturists, more sophisticated measures are likely to be needed. For example, empathy has been shown to correlate with enablement, which in turn correlates with outcome[18], and further research into other measures such as the therapeutic alliance or success of patient-practitioner interactions may be useful lines of enquiry. Our findings are consistent with a widely accepted principle underlying traditional Chinese medicine that it is not the techniques and methods used but the cultivation of the practitioner that is the key to effective practice[19]. Further qualitative work might usefully explore the ways that acupuncturists perceive that they are effective, including the ways that they cultivate themselves as practitioners. 

## Conclusions

We found little evidence of important effects on pain outcomes associated with different characteristics of acupuncture or acupuncturists, including style of acupuncture, the frequency or duration of sessions, patient-practitioner interactions or the years of experience of the acupuncturists. Increased number of needles and more sessions appear to be associated with better outcomes when comparing acupuncture to non-acupuncture controls. This suggests that the dose of acupuncture is important. Trials designed to evaluate the potentially small differences in outcome associated with different acupuncture or acupuncturist characteristics are likely to require large sample sizes. There is room for a diversity of practice in acupuncture, and no strong evidence that such diversity leads to some patients receiving sub-optimal outcomes. 

## Supporting Information

Appendix S1 **Trial and patient level information.**
(DOCX)
