# Correction: Characteristics of Acupuncture Treatment Associated with Outcome: An Individual Patient Meta-Analysis of 17,922 Patients with Chronic Pain in Randomised Controlled Trials 

**DOI:** 10.1371/annotation/23629d97-3b72-474b-9d89-c7198ba43d60

**Published:** 2013-12-03

**Authors:** Hugh MacPherson, Alexandra C. Maschino, George Lewith, Nadine E. Foster, Claudia M. Witt, Andrew J. Vickers, on behalf of the Acupuncture Trialists' Collaboration

The name of the fifth author was given incorrectly. The correct name is: Claudia M. Witt. The correct Citation is: MacPherson H, Maschino AC, Lewith G, Foster NE, Witt CM, et al. (2013) Characteristics of Acupuncture Treatment Associated with Outcome: An Individual Patient Meta-Analysis of 17,922 Patients with Chronic Pain in Randomised Controlled Trials. PLoS ONE 8(10): e77438. doi:10.1371/journal.pone.0077438. The correct abbreviation of the fifth author's name in the Author Contributions Statement is: CMW. 

