# Supplementary material for: Characteristics of Acupuncture Treatment Associated with Outcome: An Individual Patient Meta-Analysis of 17,922 Patients with Chronic Pain in Randomised Controlled Trials
Source: PLoS One. 2013 Oct 11;8(10):e77438. doi: 10.1371/journal.pone.0077438 (PMC3795671; doi:10.1371/journal.pone.0077438)
Supplement: Appendix S1 — Trial and patient level information. (DOCX) [file pone.0077438.s001.docx]

**Appendix S1. Trial level information and patient level information where analysed**.

| **Pain Type** | **Trial** | **Patient counts for those included in primary analysis** | **Result reported by author** |
| --- | --- | --- | --- |
| **Chronic headache/migraine (n=6)** | | | |
| Migraine n=2 | Linde 2005[^1^](#_ENREF_1) | **Total n=272**  **Acupuncture n=132**  **Sham**  Penetrating needle **n=76**  **No acupuncture control**  Usual care **n=64** | **Difference between groups**  **Acupuncture vs. sham: 0.0 (95% CI -0.7, 0.7) p>0.9**  **Acupuncture vs no acupuncture control: 1.4 (95% CI 0.8, 2.1) p<0.001**  **Style of acupuncture: Combination of traditional Chinese and Western**  **Point Prescription: Flexible Formula**  **Location of Needles: Both Local and Distal Points**  **Electrical stimulation allowed: No**  **Manual stimulation allowed: Yes**  **Moxibustion allowed: No**  **De Qi elicited: Yes**  **Acupuncture-specific patient practitioner interactions: No**  **Minimum years of experience required: 3**  **Maximum number of sessions: 12**  **Frequency of sessions: 1.5**  **Duration of sessions: 29**  **Number of needles used: 17** |
|  | Diener 2006[^2^](#_ENREF_2) | **Total n=794^a^**  **Acupuncture n=290**  **Sham**  Penetrating needle **n=317**  **No acupuncture control**  Guidelined care **n=187** | **Difference between groups**  **Acupuncture vs. sham: 0.57 (0.09, 1.05) p=0.021**  **Acupuncture vs no acupuncture control: 0.50 (95% CI -0.06, 1.05) p=0.4**  **Style of acupuncture: Traditional Chinese**  **Point Prescription: Flexible Formula**  **Location of Needles: Both Local and Distal Points**  **Electrical stimulation allowed: No**  **Manual stimulation allowed: Yes**  **Moxibustion allowed: No**  **De Qi elicited: Yes**  **Acupuncture-specific patient practitioner interactions: No**  **Minimum years of experience required: 2**  **Maximum number of sessions: 10**  **Frequency of sessions: 2**  **Duration of sessions: 32 (patient level data analysed)**  **Number of needles used: 16 (patient level data analysed)** |
| Tension-type headache n=2 | Melchart 2005[^3^](#_ENREF_3) | **Total n=238**  **Acupuncture n=118**  **Sham**  Penetrating needle  **n=57**  **No acupuncture control**  Usual care **n=63** | **Difference between groups**  **Acupuncture vs. sham: 0.06 (-1.2, 2.4) p=0.5**  **Acupuncture vs no acupuncture control: 5.8 (95% CI 4.0, 7.6) p<0.001**  **Difference between groups**  **Acupuncture vs. sham: 0.57 (0.09, 1.05) p=0.021**  **Acupuncture vs no acupuncture control: 0.50 (95% CI -0.06, 1.05) p=0.4**  **Style of acupuncture: Combination of traditional Chinese and Western**  **Point Prescription: Flexible Formula**  **Location of Needles: Both Local and Distal Points**  **Electrical stimulation allowed: No**  **Manual stimulation allowed: Yes**  **Moxibustion allowed: No**  **De Qi elicited: Yes**  **Acupuncture-specific patient practitioner interactions: No**  **Minimum years of experience required: 3**  **Maximum number of sessions: 12**  **Frequency of sessions: 1.5**  **Duration of sessions: 30**  **Number of needles used: 15** |
|  | Endres 2007[^4^](#_ENREF_4) | **Total n=398**  **Acupuncture n=204**  **Sham**  Penetrating needle  **n=194** | **Difference between groups**  **Acupuncture vs. sham: 1.94 (95% CI 0.69, 3.18) p=0.002**  **Style of acupuncture: Traditional Chinese**  **Point Prescription: Flexible Formula**  **Location of Needles: Both Local and Distal Points**  **Electrical stimulation allowed: No**  **Manual stimulation allowed: Yes**  **Moxibustion allowed: No**  **De Qi elicited: Yes**  **Acupuncture-specific patient practitioner interactions: No**  **Minimum years of experience required: None**  **Minimum years of experience required: 0**  **Maximum number of sessions: 10**  **Frequency of sessions: 2**  **Duration of sessions: 32**  **Number of needles used: 16** |
| Both migraine and headache  n=2 | Vickers 2004[^5^](#_ENREF_5) | **Total n=301**  **Acupuncture n=161**  **No acupuncture control**  Usual care **n=140** | **Difference between groups**  **Acupuncture vs no acupuncture control: 4.6 (95% CI 2.2, 7.0) p=0.0002**  **Style of acupuncture: Traditional Chinese**  **Point Prescription: Fully Individualized**  **Location of Needles: Both Local and Distal Points**  **Electrical stimulation allowed: No**  **Manual stimulation allowed: Yes**  **Moxibustion allowed: Yes**  **De Qi elicited: Yes**  **Acupuncture-specific patient practitioner interactions: Yes**  **Minimum years of experience required: None**  **Minimum years of experience required: 0**  **Maximum number of sessions: 24 (patient level data analysed)**  **Frequency of sessions: 1**  **Duration of sessions: 30**  **Number of needles used: Not Provided** |

| **Pain Type** | **Trial** | **Patient counts for those included in primary analysis** | **Result reported by author** |
| --- | --- | --- | --- |
|  | Jena 2008[^6^](#_ENREF_6) | **Total n=2871^b^**  **Acupuncture n=1447**  **No acupuncture control**  Usual care **n=1424** | **Difference between groups**  **Acupuncture vs no acupuncture control:** 27.9 (95% CI 25.1, 30.6) p<0.001  **Style of acupuncture: Combination of traditional Chinese and Western**  **Point Prescription: Fully Individualized**  **Location of Needles: Both Local and Distal Points**  **Electrical stimulation allowed: No**  **Manual stimulation allowed: Yes**  **Moxibustion allowed: No**  **De Qi elicited: Not Provided**  **Acupuncture-specific patient practitioner interactions: Yes**  **Minimum years of experience required: None**  **Minimum years of experience required: 0**  **Age of acupuncturists: Yes (patient level data analysed)**  **Sex of acupuncturists: Yes (patient level data analysed)**  **Maximum number of sessions: 30 (patient level data analysed)**  **Frequency of sessions: 1**  **Duration of sessions: Not Provided**  **Number of needles used: Not Provided** |

| **Pain Type** | **Trial** | **Patient counts for those included in primary analysis** | **Result reported by author** |
| --- | --- | --- | --- |
| **Non-specific Musculoskeletal**  **n=13** | | | |
| Back n=8 | Carlsson 2001[^7^](#_ENREF_7) | **Total n=27**  **Acupuncture n=21**  **Sham**  Non-needle **n=6** | **Difference between groups**  **Acupuncture vs. sham**:  Morning VAS: p=0.13 (no estimate given)  Night VAS: p=0.056 (no estimate given)  **Style of acupuncture: Combination of traditional Chinese and Western**  **Point Prescription: Flexible Formula**  **Location of Needles: Both Local and Distal Points**  **Electrical stimulation allowed: Yes**  **Manual stimulation allowed: Yes**  **Moxibustion allowed: No**  **De Qi elicited: Yes**  **Acupuncture-specific patient practitioner interactions: No**  **Minimum years of experience required: 5**  **Maximum number of sessions: 10**  **Frequency of sessions: 1**  **Duration of sessions: 20**  **Number of needles used: 16** |
|  | Cherkin 2001[^8^](#_ENREF_8) | **Total n=172^d^**  **Acupuncture n=89**  **No acupuncture control**  Non-specific advice **n=83** | **Difference between groups**  **Acupuncture vs no acupuncture control:** adjusted p=0.75 (no estimate given)  **Style of acupuncture: Traditional Chinese**  **Point Prescription: Fully Individualized**  **Location of Needles: Both Local and Distal Points**  **Electrical stimulation allowed: Yes**  **Manual stimulation allowed: Yes**  **Moxibustion allowed: Yes**  **De Qi elicited: Yes**  **Acupuncture-specific patient practitioner interactions: Yes**  **Minimum years of experience required: 3**  **Maximum number of sessions: 10**  **Frequency of sessions: 1.5**  **Duration of sessions: 28**  **Number of needles used: 12** |
|  | Kerr 2003[^9^](#_ENREF_9) | **Total n=46**  **Acupuncture n=26**  **Sham**  Non-needle **n=20** | **Difference between groups**  **Acupuncture vs. sham**: p=0.2 (no estimate given)  **Style of acupuncture: Western**  **Point Prescription: Fixed Formula**  **Location of Needles: Both Local and Distal Points**  **Electrical stimulation allowed: No**  **Manual stimulation allowed: Yes**  **Moxibustion allowed: No**  **De Qi elicited: Yes**  **Acupuncture-specific patient practitioner interactions: Yes**  **Minimum years of experience required: None**  **Minimum years of experience required: 0**  **Maximum number of sessions: 6**  **Frequency of sessions: 1**  **Duration of sessions: 30**  **Number of needles used: 11** |
|  | Brinkhaus 2006[^10^](#_ENREF_10) | **Total n=284**  **Acupuncture n=140**  **Sham**  Penetrating needle **n=70**  **No acupuncture control**  Usual care **n=74** | **Difference between groups**  **Acupuncture vs. sham**: 5.1 (95% CI −3.7, 13.9) p=0.3  **Acupuncture vs no acupuncture control:** 21.7 (95% CI 13.9, 30.0) p<0.001  **Style of acupuncture: Traditional Chinese**  **Point Prescription: Flexible Formula**  **Location of Needles: Both Local and Distal Points**  **Electrical stimulation allowed: No**  **Manual stimulation allowed: Yes**  **Moxibustion allowed: No**  **De Qi elicited: Yes**  **Acupuncture-specific patient practitioner interactions: Yes**  **Minimum years of experience required: 3**  **Age of acupuncturists: Yes (patient level data analysed)**  **Sex of acupuncturists: Yes (patient level data analysed)**  **Maximum number of sessions: 12 (patient level data analysed)**  **Frequency of sessions: 1.5**  **Duration of sessions: 28 (patient level data analysed)**  **Number of needles used: 17 (patient level data analysed)** |
|  | Thomas 2006[^11^](#_ENREF_11) | **Total n=182**  **Acupuncture n=123**  **No acupuncture control**  Usual care **n=59** | **Difference between groups**  **Acupuncture vs no acupuncture control:** 8.0 (95% CI 2.8, 13.2) p=0.003  **Style of acupuncture: Traditional Chinese**  **Point Prescription: Fully Individualized**  **Location of Needles: Both Local and Distal Points**  **Electrical stimulation allowed: Yes**  **Manual stimulation allowed: Yes**  **Moxibustion allowed: Yes**  **De Qi elicited: Yes**  **Acupuncture-specific patient practitioner interactions: Yes**  **Minimum years of experience required: 3**  **Maximum number of sessions: 10**  **Frequency of sessions: 1**  **Duration of sessions: 25**  **Number of needles used: 10** |
|  | Witt 2006[^12^](#_ENREF_12) | **Total n=2594^b^**  **Acupuncture n=1350**  **No acupuncture control**  Usual care **n=1244** | **Difference between groups**  **Acupuncture vs no acupuncture control:** 9.4 (95% CI 8.3, 10.5) p<0.001  **Style of acupuncture: Combination of traditional Chinese and Western**  **Point Prescription: Fully Individualized**  **Location of Needles: Both Local and Distal Points**  **Electrical stimulation allowed: No**  **Manual stimulation allowed: Yes**  **Moxibustion allowed: No**  **De Qi elicited: Not Provided**  **Acupuncture-specific patient practitioner interactions: Yes**  **Minimum years of experience required: None**  **Minimum years of experience required: 0**  **Age of acupuncturists: Yes (patient level data analysed)**  **Sex of acupuncturists: Yes (patient level data analysed)**  **Maximum number of sessions: 25 (patient level data analysed)**  **Frequency of sessions: 1**  **Duration of sessions: Not Provided**  **Number of needles used: Not Provided** |

| **Pain Type** | **Trial** | **Patient counts for those included in primary analysis** | **Result reported by author** |
| --- | --- | --- | --- |
|  | Haake 2007[^13^](#_ENREF_13) | **Total n=1117**^e^  **Acupuncture n=377**  **Sham**  Penetrating needle  **n=376**  **No acupuncture control**  Guidelined care **n=364** | **Difference between groups in treatment success ^h^**  **Acupuncture vs. no acupuncture control:** 20.2% (95% CI 13.4%, 26.7%) p<.001  **Acupuncture vs. sham:** 3.4% (95% CI −3.7%, 10.3%) p=0.4  **Style of acupuncture: Traditional Chinese**  **Point Prescription: Flexible Formula**  **Location of Needles: Both Local and Distal Points**  **Electrical stimulation allowed: No**  **Manual stimulation allowed: Yes**  **Moxibustion allowed: No**  **De Qi elicited: Yes**  **Acupuncture-specific patient practitioner interactions: No**  **Minimum years of experience required: 2**  **Maximum number of sessions: 10**  **Frequency of sessions: 2**  **Duration of sessions: 32 (patient level data analysed)**  **Number of needles used: 16 (patient level data analysed)** |
|  | Kennedy 2008[^14^](#_ENREF_14) | **Total n=40**  **Acupuncture n=22**  **Sham**  Needle, non-penetrating **n=18** | **Difference between groups**  **Acupuncture vs. sham**: 2.6 (95% CI −0.7, 5.9) p= 0.12  **Style of acupuncture: Western**  **Point Prescription: Flexible Formula**  **Location of Needles: Both Local and Distal Points**  **Electrical stimulation allowed: No**  **Manual stimulation allowed: Yes**  **Moxibustion allowed: No**  **De Qi elicited: Yes**  **Acupuncture-specific patient practitioner interactions: No**  **Minimum years of experience required: 10**  **Maximum number of sessions: 12**  **Frequency of sessions: 1.5**  **Duration of sessions: 30**  **Number of needles used: 11** |
| **Neck n=5** | Irnich 2001[^15^](#_ENREF_15) | **Total n=108**  **Acupuncture n=51**  **Sham**  Non-needle **n=57** | **Difference between groups**  **Acupuncture vs. sham**: 6.9 (-5.0, 18.9 ) p=0.3  **Style of acupuncture: Combination of traditional Chinese and Western**  **Point Prescription: Fully Individualized**  **Location of Needles: Both Local and Distal Points**  **Electrical stimulation allowed: No**  **Manual stimulation allowed: Yes**  **Moxibustion allowed: No**  **De Qi elicited: Yes**  **Acupuncture-specific patient practitioner interactions: No**  **Minimum years of experience required: None**  **Minimum years of experience required: 0**  **Maximum number of sessions: 5**  **Frequency of sessions: 2**  **Duration of sessions: 30**  **Number of needles used: 9** |
|  | White 2004[^16^](#_ENREF_16) | **Total n=124 ^f^**  **Acupuncture n=63**  **Sham**  Non-needle **n=61** | **Difference between groups**  **Acupuncture vs. sham:** 6.3 (95% CI 1.4, 11.3) p =0.012  **Style of acupuncture: Western**  **Point Prescription: Flexible Formula**  **Location of Needles: Both Local and Distal Points**  **Electrical stimulation allowed: No**  **Manual stimulation allowed: Yes**  **Moxibustion allowed: No**  **De Qi elicited: Yes**  **Acupuncture-specific patient practitioner interactions: No**  **Minimum years of experience required: 5**  **Maximum number of sessions: 8**  **Frequency of sessions: 2**  **Duration of sessions: 30**  **Number of needles used: 6** |
|  | Salter 2006[^17^](#_ENREF_17) | **Total n=21**  **Acupuncture n=9**  **No acupuncture control**  Usual care **n=12** | **Difference between groups**  **Acupuncture vs. no acupuncture control:** 1.75 (no confidence interval given) p = 0.8  **Style of acupuncture: Traditional Chinese**  **Point Prescription: Fully Individualized**  **Location of Needles: Both Local and Distal Points**  **Electrical stimulation allowed: Yes**  **Manual stimulation allowed: Yes**  **Moxibustion allowed: Yes**  **De Qi elicited: Yes**  **Acupuncture-specific patient practitioner interactions: Yes**  **Minimum years of experience required: 3**  **Maximum number of sessions: 10**  **Frequency of sessions: 1**  **Duration of sessions: 25**  **Number of needles used: 13** |
|  | Vas 2006[^18^](#_ENREF_18) | **Total n=123^c^**  **Acupuncture n=61**  **Sham**  Non-needle **n=62** | **Difference between groups**  **Acupuncture vs. sham**: 28.1 (95% CI 21.4, 34.7) p<0.001  **Style of acupuncture: Traditional Chinese**  **Point Prescription: Flexible Formula**  **Location of Needles: Both Local and Distal Points**  **Electrical stimulation allowed: No**  **Manual stimulation allowed: Yes**  **Moxibustion allowed: No**  **De Qi elicited: Yes**  **Acupuncture-specific patient practitioner interactions: No**  **Minimum years of experience required: 3**  **Maximum number of sessions: 5**  **Frequency of sessions: 1.5**  **Duration of sessions: 30**  **Number of needles used: 10** |
|  | Witt 2006[^19^](#_ENREF_19) | **Total n=3162^b^**  **Acupuncture n=1618**  **No acupuncture control**  Usual care **n=1544** | **Difference between groups**  **Acupuncture vs no acupuncture control:** 12.3 (95% CI 11.3, 13.3) p < 0.001  **Style of acupuncture: Combination of traditional Chinese and Western**  **Point Prescription: Fully Individualized**  **Location of Needles: Both Local and Distal Points**  **Electrical stimulation allowed: No**  **Manual stimulation allowed: Yes**  **Moxibustion allowed: No**  **De Qi elicited: Not Provided**  **Acupuncture-specific patient practitioner interactions: Yes**  **Minimum years of experience required: None**  **Minimum years of experience required: 0**  **Age of acupuncturists: Yes (patient level data analysed)**  **Sex of acupuncturists: Yes (patient level data analysed)**  **Maximum number of sessions: 30 (patient level data analysed)**  **Frequency of sessions: 1**  **Duration of sessions: Not Provided**  **Number of needles used: Not Provided** |

| **Pain Type** | **Trial** | **Patient counts for those included in primary analysis** | **Result reported by author** |
| --- | --- | --- | --- |
| **Osteoarthritis**  **n=7** | | | |
|  | Berman 2004[^20^](#_ENREF_20) | **Total n=391**  **Acupuncture n=142**  **Sham**  Both penetrating and non-penetrating needles **n=141**  **No acupuncture control**  Non-specific advice **n=108** | **Difference between groups**  **Acupuncture vs. sham**: 0.87 (95% CI 0.16, 1.58) p=0.003  **Acupuncture vs no acupuncture control:** (not given)  **Style of acupuncture: Traditional Chinese**  **Point Prescription: Fixed Formula**  **Location of Needles: Both Local and Distal Points**  **Electrical stimulation allowed: Yes**  **Manual stimulation allowed: Yes**  **Moxibustion allowed: No**  **De Qi elicited: Yes**  **Acupuncture-specific patient practitioner interactions: No**  **Minimum years of experience required: 2**  **Maximum number of sessions: 23**  **Frequency of sessions: .88**  **Duration of sessions: 20**  **Number of needles used: 11** |
|  | Vas 2004[^21^](#_ENREF_21) | **Total n=88**  **Acupuncture n=47**  **Sham**  Non-penetrating needle  **n=41** | **Difference between groups**  **Acupuncture vs. sham**: 4.7 (95% CI 2.9, 6.5) p<0.001  **Style of acupuncture: Traditional Chinese**  **Point Prescription: Flexible Formula**  **Location of Needles: Both Local and Distal Points**  **Electrical stimulation allowed: Yes**  **Manual stimulation allowed: Yes**  **Moxibustion allowed: No**  **De Qi elicited: Yes**  **Acupuncture-specific patient practitioner interactions: No**  **Minimum years of experience required: 3**  **Maximum number of sessions: 13**  **Frequency of sessions: 1**  **Duration of sessions: 30**  **Number of needles used: 8** |
|  | Witt 2005[^22^](#_ENREF_22) | **Total n=286**  **Acupuncture n=145**  **Sham**  Penetrating needle  **n=73**  **No acupuncture control**  Usual care **n=67** | **Difference between groups**  **Acupuncture vs. sham**: 8.8 (95% CI 4.2, 13.5) p<0.001  **Acupuncture vs no acupuncture control:** 22.7 (95% CI 17.9, 27.5) p<0.001  **Style of acupuncture: Combination of traditional Chinese and Western**  **Point Prescription: Flexible Formula**  **Location of Needles: Both Local and Distal Points**  **Electrical stimulation allowed: No**  **Manual stimulation allowed: Yes**  **Moxibustion allowed: No**  **De Qi elicited: Yes**  **Acupuncture-specific patient practitioner interactions: Yes**  **Minimum years of experience required: 3**  **Age of acupuncturists: Yes (patient level data analysed)**  **Sex of acupuncturists: Yes (patient level data analysed)**  **Maximum number of sessions: 12 (patient level data analysed)**  **Frequency of sessions: 1.5**  **Duration of sessions: 29 (patient level data analysed)**  **Number of needles used: 17 (patient level data analysed)** |
|  | Scharf 2006[^23^](#_ENREF_23) | **Total n=1085^b^**  **Acupuncture n=318**  **Sham**  Penetrating needle **n=360**  **No acupuncture control**  Ancillary care **n=307** | **Difference between groups**  **Acupuncture vs sham:** 0.3 (95% CI -0.05, 0.59) (no p value given)  **Acupuncture vs no acupuncture control:** 1.0 (95% CI 0.71, 1.38) (no p value given)  **Style of acupuncture: Traditional Chinese**  **Point Prescription: Flexible Formula**  **Location of Needles: Both Local and Distal Points**  **Electrical stimulation allowed: No**  **Manual stimulation allowed: Yes**  **Moxibustion allowed: No**  **De Qi elicited: Yes**  **Acupuncture-specific patient practitioner interactions: No**  **Minimum years of experience required: 2**  **Maximum number of sessions: 10**  **Frequency of sessions: 1.67**  **Duration of sessions: 30 (patient level data analysed)**  **Number of needles used: 13 (patient level data analysed)** |
|  | Witt 2006[^24^](#_ENREF_24) | **Total n=579^b^**  **Acupuncture n=300**  **No acupuncture control**  Usual care **n=279** | **Difference between groups**  **Acupuncture vs no acupuncture control:** 16.7 (SEM 1.4) p<0.001  **Style of acupuncture: Combination of traditional Chinese and Western**  **Point Prescription: Fully Individualized**  **Location of Needles: Both Local and Distal Points**  **Electrical stimulation allowed: No**  **Manual stimulation allowed: Yes**  **Moxibustion allowed: No**  **De Qi elicited: Not Provided**  **Acupuncture-specific patient practitioner interactions: Yes**  **Minimum years of experience required: None**  **Minimum years of experience required: 0**  **Age of acupuncturists: Yes (patient level data analysed)**  **Sex of acupuncturists: Yes (patient level data analysed)**  **Maximum number of sessions: 15 (patient level data analysed)**  **Frequency of sessions: 1**  **Duration of sessions: Not Provided**  **Number of needles used: Not Provided** |
|  | Foster 2007[^25^](#_ENREF_25) | **Total n=325**  **Acupuncture n=108**  **Sham**  Non-penetrating needle **n=112**  **No acupuncture control**  Ancillary care **n=105** | **Difference between groups**  **Acupuncture vs sham:** (not given)  **Acupuncture vs no acupuncture control:** 0.08 (95% CI −1.0, 0.9) p= 0.9  **Style of acupuncture: Traditional Chinese**  **Point Prescription: Flexible Formula**  **Location of Needles: Both Local and Distal Points**  **Electrical stimulation allowed: No**  **Manual stimulation allowed: Yes**  **Moxibustion allowed: No**  **De Qi elicited: Yes**  **Acupuncture-specific patient practitioner interactions: Yes**  **Minimum years of experience required: None**  **Minimum years of experience required: 0**  **Maximum number of sessions: 9 (patient level data analysed)**  **Frequency of sessions: 1**  **Duration of sessions: 30**  **Number of needles used: 8** |

| **Pain Type** | **Trial** | **Patient counts for those included in primary analysis** | **Result reported by author** |
| --- | --- | --- | --- |
|  | Williamson 2007[^26^](#_ENREF_26) | **Total n=121^c^**  **Acupuncture n=60**  **No acupuncture control**  Non-specific advice **n=61** | **Difference between groups**  **Acupuncture vs no acupuncture control**: 3.5 (95% CI 0.66, 6.33) Bonferroni p=0.016  **Style of acupuncture: Western**  **Point Prescription: Flexible Formula**  **Location of Needles: Both Local and Distal Points**  **Electrical stimulation allowed: No**  **Manual stimulation allowed: Yes**  **Moxibustion allowed: No**  **De Qi elicited: Yes**  **Acupuncture-specific patient practitioner interactions: No**  **Minimum years of experience required: None**  **Minimum years of experience required: 0**  **Maximum number of sessions: 6**  **Frequency of sessions: 1**  **Duration of sessions: 20**  **Number of needles used: 9** |

| **Pain Type** | **Trial** | **Patient counts for those included in primary analysis** | **Result reported by author** |
| --- | --- | --- | --- |
| **Specific shoulder (n=3)** | | | |
|  | Kleinhenz 1999[^27^](#_ENREF_27) | **Total n=45**  **Acupuncture n=22**  **Sham**  Non-penetrating needle  **n=23** | **Difference between groups**  **Acupuncture vs. sham**: (no estimate given) (95% CI 2.3, 19.4) p=0.001  **Style of acupuncture: Traditional Chinese**  **Point Prescription: Flexible Formula**  **Location of Needles: Both Local and Distal Points**  **Electrical stimulation allowed: No**  **Manual stimulation allowed: Yes**  **Moxibustion allowed: No**  **De Qi elicited: Yes**  **Acupuncture-specific patient practitioner interactions: No**  **Minimum years of experience required: None**  **Minimum years of experience required: 0**  **Maximum number of sessions: 8**  **Frequency of sessions: 2**  **Duration of sessions: 20**  **Number of needles used: 10** |
|  | Guerra de Hoyos 2004[^28^](#_ENREF_28) | **Total n=110**  **Acupuncture n=55**  **Sham**  Non-penetrating needle  **n=55** | **Difference between groups**  **Acupuncture vs. sham**: 2.0 (95% CI 1.2, 2.9) p<0.0005  **Style of acupuncture: Traditional Chinese**  **Point Prescription: Fixed Formula**  **Location of Needles: Both Local and Distal Points**  **Electrical stimulation allowed: Yes**  **Manual stimulation allowed: Yes**  **Moxibustion allowed: No**  **De Qi elicited: Yes**  **Acupuncture-specific patient practitioner interactions: No**  **Minimum years of experience required: None**  **Minimum years of experience required: 0**  **Maximum number of sessions: 7**  **Frequency of sessions: 1**  **Duration of sessions: 15**  **Number of needles used: 7** |
|  | Vas 2008[^29^](#_ENREF_29) | **Total n=425^c^**  **Acupuncture n=205**  **Sham**  Non-needle **n=220** | **Difference between groups**  **Acupuncture vs. sham**:6.0 (95% CI 3.2, 8.8) p<0.001  **Style of acupuncture: Traditional Chinese**  **Point Prescription: Fixed Formula**  **Location of Needles: Distal Points Only**  **Electrical stimulation allowed: No**  **Manual stimulation allowed: Yes**  **Moxibustion allowed: No**  **De Qi elicited: Yes**  **Acupuncture-specific patient practitioner interactions: No**  **Minimum years of experience required: .5**  **Maximum number of sessions: 3**  **Frequency of sessions: 1**  **Duration of sessions: 30**  **Number of needles used: 1** |

**Notes**

Ancillary care: Programme of care received by both acupuncture and non-acupuncture groups (e.g. trial comparing physiotherapy plus acupuncture to physiotherapy alone).

Usual care: Protocol did not specify treatments received in control group (e.g. trials with “waiting list controls”).

Non-specific advice: Patients in control group receive general advice and support (“attention control”).

Guidelined care: Patients in control group received care according to national guidelines.

a These differ from the patient counts in the forest plot of the primary research^30^. Authors confirmed this was an error on their part and have published an *erratum*.

b Patient counts lower in the forest plots of the primary research^30^ due to missing baseline scores for some patients.

c Patient counts lower in the forest plots of the primary research^30^ as number reported in paper includes imputed data.

d One person in the no acupuncture control group was missing Roland Morris Disability Questionnaire data but this was not reported in the paper

e Lower patient counts in our analyses of the primary research^30^ are due to missing randomization stratification variables: baseline Von Korff, chronification, fear avoidance belief, levels of activity, patient expectations,

or trial center.

f We averaged weeks 4, 5, & 6 to get a 1 month score.

g These numbers were taken from data provided, can only be estimated from what is given in the paper

h Values are given as percentage of patients (95% confidence interval). Success was defined as 33% improvement or better on 3 pain-related items on the CPGS.

i Pain relief >= 50%

j We combine the individualized and standardized acupuncture estimates in our analyses.

**References**

**1.** Linde K, Streng A, Jurgens S, et al. Acupuncture for patients with migraine: a randomized controlled trial. *JAMA.* May 4 2005;293(17):2118-2125.

**2.** Diener HC, Kronfeld K, Boewing G, et al. Efficacy of acupuncture for the prophylaxis of migraine: a multicentre randomised controlled clinical trial. *Lancet Neurol.* Apr 2006;5(4):310-316.

**3.** Melchart D, Streng A, Hoppe A, et al. Acupuncture in patients with tension-type headache: randomised controlled trial. *BMJ.* Aug 13 2005;331(7513):376-382.

**4.** Endres HG, Bowing G, Diener HC, et al. Acupuncture for tension-type headache: a multicentre, sham-controlled, patient-and observer-blinded, randomised trial. *J Headache Pain.* Oct 2007;8(5):306-314.

**5.** Vickers AJ, Rees RW, Zollman CE, et al. Acupuncture for chronic headache in primary care: large, pragmatic, randomised trial. *BMJ.* Mar 27 2004;328(7442):744.

**6.** Jena S, Witt CM, Brinkhaus B, Wegscheider K, Willich SN. Acupuncture in patients with headache. *Cephalalgia.* Sep 2008;28(9):969-979.

**7.** Carlsson CP, Sjolund BH. Acupuncture for chronic low back pain: a randomized placebo-controlled study with long-term follow-up. *Clin J Pain.* Dec 2001;17(4):296-305.

**8.** Cherkin DC, Eisenberg D, Sherman KJ, et al. Randomized trial comparing traditional Chinese medical acupuncture, therapeutic massage, and self-care education for chronic low back pain. *Arch Intern Med.* Apr 23 2001;161(8):1081-1088.

**9.** Kerr DP, Walsh DM, Baxter D. Acupuncture in the management of chronic low back pain: a blinded randomized controlled trial. *Clin J Pain.* Nov-Dec 2003;19(6):364-370.

**10.** Brinkhaus B, Witt CM, Jena S, et al. Acupuncture in patients with chronic low back pain: a randomized controlled trial. *Arch Intern Med.* Feb 27 2006;166(4):450-457.

**11.** Thomas KJ, MacPherson H, Thorpe L, et al. Randomised controlled trial of a short course of traditional acupuncture compared with usual care for persistent non-specific low back pain. *BMJ.* Sep 23 2006;333(7569):623.

**12.** Witt CM, Jena S, Selim D, et al. Pragmatic randomized trial evaluating the clinical and economic effectiveness of acupuncture for chronic low back pain. *Am J Epidemiol.* Sep 1 2006;164(5):487-496.

**13.** Haake M, Muller HH, Schade-Brittinger C, et al. German Acupuncture Trials (GERAC) for chronic low back pain: randomized, multicenter, blinded, parallel-group trial with 3 groups. *Arch Intern Med.* Sep 24 2007;167(17):1892-1898.

**14.** Kennedy S, Baxter GD, Kerr DP, Bradbury I, Park J, McDonough SM. Acupuncture for acute non-specific low back pain: a pilot randomised non-penetrating sham controlled trial. *Complement Ther Med.* Jun 2008;16(3):139-146.

**15.** Irnich D, Behrens N, Molzen H, et al. Randomised trial of acupuncture compared with conventional massage and "sham" laser acupuncture for treatment of chronic neck pain. *BMJ.* Jun 30 2001;322(7302):1574-1578.

**16.** White P, Lewith G, Prescott P, Conway J. Acupuncture versus placebo for the treatment of chronic mechanical neck pain: a randomized, controlled trial. *Ann Intern Med.* Dec 21 2004;141(12):911-919.

**17.** Salter GC, Roman M, Bland MJ, MacPherson H. Acupuncture for chronic neck pain: a pilot for a randomised controlled trial. *BMC Musculoskelet Disord.* 2006;7:99.

**18.** Vas J, Perea-Milla E, Mendez C, et al. Efficacy and safety of acupuncture for chronic uncomplicated neck pain: a randomised controlled study. *Pain.* Dec 15 2006;126(1-3):245-255.

**19.** Witt CM, Jena S, Brinkhaus B, Liecker B, Wegscheider K, Willich SN. Acupuncture for patients with chronic neck pain. *Pain.* Jun 13 2006.

**20.** Berman BM, Lao L, Langenberg P, Lee WL, Gilpin AM, Hochberg MC. Effectiveness of acupuncture as adjunctive therapy in osteoarthritis of the knee: a randomized, controlled trial. *Ann Intern Med.* Dec 21 2004;141(12):901-910.

**21.** Vas J, Mendez C, Perea-Milla E, et al. Acupuncture as a complementary therapy to the pharmacological treatment of osteoarthritis of the knee: randomised controlled trial. *BMJ.* Nov 20 2004;329(7476):1216.

**22.** Witt C, Brinkhaus B, Jena S, et al. Acupuncture in patients with osteoarthritis of the knee: a randomised trial. *Lancet.* Jul 9-15 2005;366(9480):136-143.

**23.** Scharf HP, Mansmann U, Streitberger K, et al. Acupuncture and knee osteoarthritis: a three-armed randomized trial. *Ann Intern Med.* Jul 4 2006;145(1):12-20.

**24.** Witt CM, Jena S, Brinkhaus B, Liecker B, Wegscheider K, Willich SN. Acupuncture in patients with osteoarthritis of the knee or hip: a randomized, controlled trial with an additional nonrandomized arm. *Arthritis Rheum.* Nov 2006;54(11):3485-3493.

**25.** Foster NE, Thomas E, Barlas P, et al. Acupuncture as an adjunct to exercise based physiotherapy for osteoarthritis of the knee: randomised controlled trial. *BMJ.* Sep 1 2007;335(7617):436.

**26.** Williamson L, Wyatt MR, Yein K, Melton JT. Severe knee osteoarthritis: a randomized controlled trial of acupuncture, physiotherapy (supervised exercise) and standard management for patients awaiting knee replacement. *Rheumatology (Oxford).* Sep 2007;46(9):1445-1449.

**27.** Kleinhenz J, Streitberger K, Windeler J, Gussbacher A, Mavridis G, Martin E. Randomised clinical trial comparing the effects of acupuncture and a newly designed placebo needle in rotator cuff tendinitis. *Pain.* Nov 1999;83(2):235-241.

**28.** Guerra de Hoyos JA, Andres Martin Mdel C, Bassas y Baena de Leon E, et al. Randomised trial of long term effect of acupuncture for shoulder pain. *Pain.* Dec 2004;112(3):289-298.

**29.** Vas J, Ortega C, Olmo V, et al. Single-point acupuncture and physiotherapy for the treatment of painful shoulder: a multicentre randomized controlled trial. *Rheumatology (Oxford).* Jun 2008;47(6):887-893.

**30**. Vickers AJ, Cronin AM, Maschino AC, Lewith G, MacPherson H, Foster NE, et al. Acupuncture for Chronic Pain: Individual Patient Data Meta-analysis. *Arch Intern Med* 2012 Sep 10;1-10.
